# Technical Success after Transcatheter Aortic Valve Replacement for Bicuspid versus Tricuspid Aortic Stenosis

**DOI:** 10.3390/jcm12010343

**Published:** 2023-01-01

**Authors:** Hanyi Dai, Jiaqi Fan, Yuxin He, Jun Chen, Dao Zhou, Abuduwufuer Yidilisi, Xinrui Qi, Ranxi Li, Xianbao Liu, Jian’an Wang

**Affiliations:** Department of Cardiology, The Second Affiliated Hospital Zhejiang University School of Medicine, Hangzhou 310009, China

**Keywords:** transcatheter aortic valve replacement, bicuspid aortic stenosis, self-expanding valve, transfemoral approach, technical success

## Abstract

Background: Comparative data of the Valve Academic Research Consortium (VARC-3)-defined technical success between bicuspid versus tricuspid aortic stenosis (AS) remain lacking. Aims: We sought to compare the technical success and other clinical outcomes between patients with bicuspid and tricuspid AS receiving transcatheter aortic valve replacement. Methods: A registration-based analysis was performed for 402 patients (211 and 191 cases of bicuspid and tricuspid AS, respectively). The primary outcome was VARC-3-defined technical success. Additional analysis was performed to assess outcomes for up to one year between the two groups. Results: Bicuspid AS patients tended to be younger (74 years vs. 77 years; *p* < 0.001) with a lower Society of Thoracic Surgeons score (4.4% vs. 5.4%; *p* = 0.003). Bicuspid AS patients showed a lower prevalence of hypertension and peripheral vascular diseases. Technical failure was encountered in 17.7% of these patients, driven primarily by the high incidence of second valve implantation. The technical success rates were comparable between the bicuspid and tricuspid AS groups (82.5% vs. 82.2%, *p* = 0.944). Chronic kidney disease (CKD) and larger sinotubular junctional diameter (STJ) were identified as predictors of technical failure, whereas CKD, impaired left ventricular ejection fraction (LVEF), along with larger STJ, were predictors of cardiac technical failure. Technical failure was associated with an increased risk of all-cause mortality at 30 days and 1 year, as evidenced by the Cox multivariable analysis. Conclusions: No significant differences were observed in the technical success rates and most clinical outcomes between the bicuspid and tricuspid AS groups. Technical failure conferred an increased risk for both 30-day and 1-year all-cause mortalities.

## 1. Introduction

Owing to the safety, efficacy, and patient acceptability, transcatheter aortic valve replacement (TAVR) has emerged as an established treatment strategy for cases of severe aortic stenosis (AS) irrespective of the surgical risk stratification [1,2]. Due to the expansion of TAVR technology toward younger and low-risk AS patients [3,4], the proportion of potential TAVR candidates with bicuspid AS is on the rise. Therefore, TAVR application for the treatment of bicuspid AS requires particular attention given its complicated morphological characteristics [5].

The updated Valve Academic Research Consortium-3 (VARC-3) for aortic valve clinical research recommends technical success as a novel clinical composite endpoint, substantially different from device success defined in VARC-2 [6,7]. The characteristics of technical failure in a large prospective TAVR registry were recently reported, demonstrating that technical failure was associated with poor clinical outcomes at one year [8]. However, the proportion of TAVR candidates with bicuspid AS in the above study was as low as 5.4%, and no subgroup analysis was conducted [8]. Though Yoon et al. noted that bicuspid AS patients had similar satisfactory survival rates as TAV patients, but with lower VARC-2 device success [9], little is known about the differences in technical success between the bicuspid and tricuspid AS population based on VARC-3 criteria. Therefore, we aimed to investigate the incidence, predictors, and impact on the prognosis of technical failure in a cohort comprising nearly 50% of patients with bicuspid AS.

## 2. Methods

Study Design and Population Severe AS patients treated with transfemoral TAVR at a high-volume tertiary center between May 2013 and June 2021 were consecutively enrolled. Severe AS was defined as an effective orifice area of ≤1 cm^2^ with a mean transvalvular gradient of ≥40 mmHg, or a maximum transvalvular velocity of ≥4 m/s. The multidisciplinary heart team determined the eligibility of all TAVR candidates before enrollment. Patients with a quadricuspid aortic valve, pure aortic regurgitation, prior aortic valve replacement, and those undergoing emergency procedures were all excluded. The first-generation self-expanding devices whose delivery system could not be repositioned and retrieved were implanted in all TAVR recipients, including VenusA Valve (Venus Medtech, Hangzhou, China), CoreValve (Medtronic, Minneapolis, Minnesota), VitaFlow Vavle (MicroPort, Shanghai, China), and TaurusOne Valve (Peijia Medical, Suzhou, China). Hangzhou Solution was used as prosthesis sizing selection guide in bicuspid AS patients. This research protocol was approved by the Institutional Ethics Committee and complied with the Declaration of Helsinki. All participants provided informed consent for TAVR procedures and their relevant data were anonymized.

Preoperative loading of dual antiplatelet therapy is not mandatory unless severe coronary artery stenosis identification before the TAVR procedure. Left ventricular ejection fraction (LVEF) ≤ 50% is defined as impaired LVEF. An estimated glomerular filtration rate < 60 mL/min/1.73 m^2^ suggested chronic kidney disease (CKD) [10]. Surgery or intervention for femoral artery stenosis was performed after a comprehensive evaluation.

Bicuspid Aortic Valve All patients underwent multi-detected computed tomography (MDCT) scanning, which is considered to be the gold standard for determining the classification of bicuspid aortic valve stenosis. Two independent physicians (H.Y.D. and D.Z.) classified bicuspid aortic valve morphology using the 3mensio software (3mensio Medical Imaging BV, Bilthoven, The Netherlands). Bicuspid aortic valve morphology was classified into type 0, type 1 and type 2 following the bicuspid aortic valve classification proposed by Sievers et al. [11]. Type 0 indicated the presence of only two malformed functional cusps without fused raphe. Type 1 represents two adjacent underdeveloped cusps, one fully developed cusp, and one raphe, while type 2 represents two underdeveloped cusps with two raphes.

Study Endpoint The primary outcome was updated VARC-3-defined composite outcome technical success. Additional analyses were performed to assess outcomes between the two groups for up to one year. By the VARC-3 definition, the composite endpoint technical success was assessed at the exit from the catheterization room [6]. Specifically, technical success included the following: (1) freedom from procedural mortality; (2) successful access, delivery, and retrieval of the device system; (3) correct valve positioning, with no need for second valve implantation, and (4) the absence of surgery or intervention related to the device, a major vascular or access-related or cardiac structural complication. Failure to comply with any of the above-mentioned items is considered a technical failure. Specifically, technical failure was categorized into vascular technical failure limited to vascular complications or cardiac technical failure including all other criteria. A 30-day and 1-year follow-up was completed for all patients either by face-to-face assessment or telephone interviews, and the relevant data were recorded.

Statistical Analysis Continuous variables following a normal distribution are presented as mean ± standard deviation or median (25th, 75th percentiles) for the Shapiro–Wilk test. Normally distributed variables were compared using Student’s *t*-test whereas non-normally distributed variables were compared using the Mann–Whitney U test. Categorical variables were expressed as frequency (percentages) and compared using Pearson’s chi-squared test or Fisher exact test. Univariate and multivariate logistic regression analyses were conducted to identify potential risk predictors for technical failure. Kaplan–Meier method and the log-rank test were used for visualizing and comparing cumulative survival probabilities in the groups. The relationship between technical failure and all-cause mortality was determined using time-independent Cox proportional-hazards models. The simultaneous effects of risk factors were also estimated using Cox proportional hazards models. All statistical tests were performed using the SPSS software (IBM SPSS version 25.0, New York, NY, USA). Statistically significant variables were two-tailed *p*-values less than 0.05 for all tests.

## 3. Results

A total of 402 patients who underwent transfemoral TAVR procedures were enrolled in the study. Among them, 229 (52.5%) were cases of bicuspid AS and the remaining 191 (47.5%) were of tricuspid AS. The baseline demographics and clinical characteristics of the study population are presented in Table 1. Over half of the participants (57.7%) were males. As expected, the baseline characteristics were unbalanced, and multiple differences existed between the two groups. For instance, compared to tricuspid AS patients, bicuspid AS patients tended to be younger (74 years (interquartile range (IQR): 69 to 79 years) vs. 77 years (IQR: 71 to 82 years); *p* < 0.001). Bicuspid AS patients also had a lower Society of Thoracic Surgeons predicted risk of 30-day mortality (STS-PROM) (4.4 (IQR: 2.6 to 7.6) vs. 5.4 (IQR: 3.4 to 9.3); *p* = 0.003) and showed lower incidences of hypertension and peripheral vascular diseases. Previous medical history was similar between the two groups except for the lower prevalence of peripheral vascular diseases (9.0% vs.19.9%; *p* = 0.002) in the bicuspid AS group.

Typically at baseline, bicuspid AS patients showed higher mean transvalvular gradient (53.0 mmHg (IQR: 43.0 to 71.0 mmHg) vs. 50.5 mmHg (IQR: 41.0 to 64.0 mmHg); *p* = 0.013) and maximal transvalvular velocity (4.8 m/s (IQR: 4.3 to 5.5 m/s] vs. 4.7 m/s (IQR:4.3 to 5.2 m/s); *p* = 0.056) but smaller aortic valve area (0.6 cm^2^ (IQR: 0.4 to 0.7 cm^2^) vs. 0.7 cm^2^ (IQR: 0.5 to 0.8 cm^2^), *p* < 0.001). For the anatomy of the aortic root measured on MDCT scans, bicuspid AS patients were more likely to present with larger aortic sinuses, ascending aortopathy, and heavier annulus calcium. All dimensions of the aortic root with exception of the annular perimeter-derived diameter in the bicuspid AS group were significantly larger than those in the tricuspid AS group. Specifically, bicuspid AS patients had larger STJ diameter (31.3 mm (IQR: 29.1 to 34.1 mm) vs. 29.3 mm (IQR: 26.2 to 32.2 mm); *p* < 0.001) and larger ascending aorta diameter at 4 cm (39.0 mm (IQR: 36.3 to 42.0 mm) vs. 35.7 mm (IQR: 33.1 to 38.8 mm); *p* < 0.001). Bicuspid AS patients had higher left main coronary artery ostium (14.9 mm (IQR: 12.9 to 18.1 mm)) vs. 13.3 mm (IQR: 11.3 to 15.6 mm); *p* < 0.001) and more severe calcification (57.4% vs. 46.6%; *p* = 0.030).

Notably, all TAVR procedures were performed via transfemoral access using first-generation self-expanding devices. Procedural characteristics and results are summarized in Table 2. Balloon pre-dilatation, routinely conducted at our center, was performed in 98.7% of these patients. Bicuspid AS patients tended toward a higher prevalence of balloon pre-dilatation (99.5% vs. 97.4%, *p* = 0.077) and post-dilation (68.2% vs. 55.0%, *p* = 0.006). The incidences of most procedural outcomes were comparable between the two groups; however, patients with bicuspid AS had a lower incidence of permanent pacemaker implantation (6.6% vs. 14.7%, *p* = 0.009).

For 30-day clinical outcomes (Table 3), the all-cause mortality rate was similar between the bicuspid and tricuspid AS groups (2.4% vs. 2.1%, *p* = 1.000). Similar outcomes were observed between the two groups for most clinical outcomes except for a lower incidence of permanent pacemaker implantation in the bicuspid AS population. Over a one-year follow-up period, 11 (5.2%) and 20 (10.5%) patients with bicuspid and tricuspid AS died, respectively. There were no significant differences in cumulative all-cause mortality, cardiovascular mortality, or non-cardiovascular mortality between the two groups at one-year follow-up.

Based on the VARC-3 criteria, 71 cases of technical failure (17.7%) were recorded and there were no differences between the bicuspid and tricuspid AS groups (82.5% vs. 82.2%; *p* = 0.944). Cardiac technical failure and vascular technical failure occurred in 53 patients (13.2%) and 23 patients (5.7%), respectively. The detailed reasons for technical failure are reported in Table 4. An additional logistic regression analysis was performed to determine the predictors of a technical failure (Table 5). Bicuspid AS was not associated with an increased risk of technical failure, as evidenced by univariable logistic analysis. Additionally, in bicuspid AS-adjusted multivariable regression analysis, CKD and STJ diameter ≥ 31.0 mm were identified as the predictors of technical failure whereas CKD, impaired LVEF along with an STJ diameter ≥ 31.0 mm remained independent predictive factors of cardiac technical failure.

**Figure 1:**
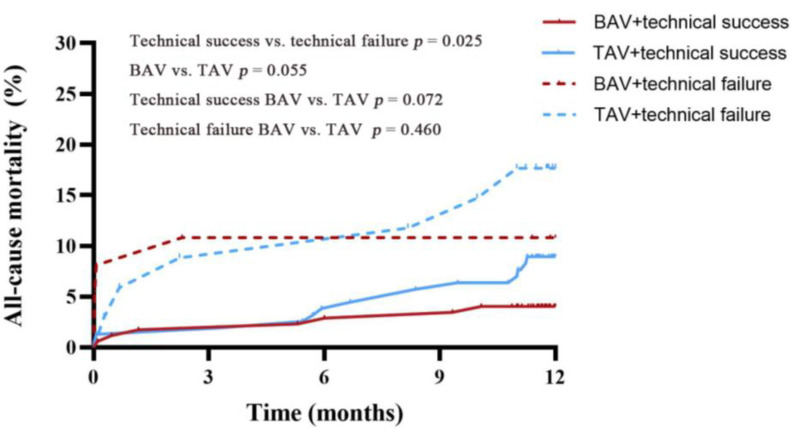
Kaplan–Meier curves demonstrating all-cause mortality at 1 year in BAV and TAV groups, stratified by technical success or technical failure. *p*-values were calculated using log rank tests. BAV = bicuspid aortic valve; TAV = tricuspid aortic valve.

Cox proportional hazard regression model was performed for detecting the independent predictive factors of all-cause mortality. As Cox multivariate analysis showed (Table 6 and Table 7), bicuspid AS was not related to 30-day mortality (*p* = 0.716) or 1-year mortality (*p* = 0.353). Technical failure (*p* = 0.031), higher STS scores (*p* < 0.001), STJ diameter ≥ 31.0 mm (*p* = 0.019) were associated with 30-day mortality, while technical failure (*p* = 0.021) and higher STS scores (*p* < 0.001) were independent predictive factors of 1-year mortality. Kaplan–Meier estimates of all-cause mortality at 1 year are presented in Figure 1. BAV was not associated with significantly increased all-cause mortality (*p* = 0.055). This finding persisted in technical success (*p* = 0.072) and technical failure (*p* = 0.460) subgroup analyses.

## 4. Discussions

We sought to compare the technical success rates after TAVR between bicuspid and tricuspid AS patients. The principal findings of the present study were as follows: (1) technical success was achieved in 82.3% of patients treated with TAVR, similar between the bicuspid and tricuspid AS groups. (2) CKD and STJ diameter ≥ 31.0 mm were independently associated with an increased risk of technical failure, whereas CKD, impaired LVEF, and STJ diameter ≥ 31.0 mm were associated with cardiac technical failure. (3) Technical failure was an independent predictive factor of 30-day and 1-year all-cause mortality.

Recently published VARC-3 introduced a brand-new composite endpoint technical success. In contrast to the success of VARC-2 devices, updated VARC-3-defined technical success does not consider hemodynamics and can be evaluated immediately at the time of leaving the catheterization laboratory [6,7]. Nowadays, this standardized concept has been widely employed in designing studies and exerts a significant impact on outcomes up to 1 year after TAVR [8].

In the retrospective analysis of the Bern TAVR registry, 88.5% of patients achieved technical success after TAVR with contemporary devices [8]. Technical failure consists of about one-quarter of cardiac technical failure and three-quarters of vascular technical failure [8]. Nevertheless, 17.7% of patients suffered from technical failure in our study, which is higher than that reported in previous studies [8,12]. The relatively high rate of technical failure in our analysis may be related to that all patients treated with first-generation TAVR systems. Moreover, the reasons of technical failure comprised over two-thirds of cardiac technical failure and one-third of vascular technical failure, which was not consistent with that of the Bern TAVR registry. The first-generation self-expanding valves are still the most commonly used devices in TAVR procedures. The first-generation valves were associated with more frequent moderate or greater paravalvular leakage requiring the implantation of a second prothesis, which could be translated into higher cardiac technical failure in our analysis.

Bicuspid aortic valve is one of the most common congenital heart valve diseases. As TAVR gradually expands toward lower risk and younger patients, the number of TAVR procedures performed in bicuspid AS patients keep rising [13,14]. Bicuspid AS is characterized by challenging anatomical characteristics (including elliptical and larger annulus, asymmetrical leaflets, calcified raphes, and concomitant aortopathy), making the device frame exert an asymmetrical radial force on the native annulus, ultimately leading to paravalvular leakage [5,15]. Several studies have suggested that bicuspid AS patients after TAVR have worse outcomes, especially poor hemodynamics, compared to tricuspid AS patients [16]. Thus, bicuspid AS cases were excluded from the landmark TAVR clinical trials, although other studies have confirmed comparable clinical benefits between the bicuspid and tricuspid AS population [9,17,18]. In our analysis, baseline characteristics were heterogeneous between the bicuspid and tricuspid AS groups, with lower comorbidity burden and risk profiles in the bicuspid AS population. These clinical results after TAVR among bicuspid AS patients were comparable with tricuspid AS patients, which is consistent with the previous study. There was a trend toward a lower incidence of permanent pacemaker implantation in cases of bicuspid AS, which might be associated with the higher prosthesis implantation depth and effective supra-annular sizing strategies [18]. Due to the lack of clinical data comparing bicuspid AS and tricuspid AS based on VARC-3 criteria, a significant knowledge gap exists in whether bicuspid AS cases have lower technical success rates than their tricuspid AS counterparts. The proportion of bicuspid AS patients in our analysis was substantially higher compared to previous reports based on the VARC-3 definition [8]. The equivalent incidence of technical success between the two groups suggested that TAVR might be a safe and feasible therapeutic option for all patients.

With a rapid increase in TAVR recommendations, elucidating technical failure-related predictors is pivotal for selecting the most appropriate recipients and optimizing TAVR outcomes. Similar to previously established risk algorithms specific for outcomes after TAVR, CKD, and STJ diameter ≥ 31.0 mm were identified as significant predictors of technical failure whereas CKD, impaired LVEF, and STJ diameter ≥ 31.0 mm were predictors of cardiac technical failure. Patients with CKD had a higher risk of all-cause and cardiovascular mortality than those without CKD [19]. CKD can exacerbate age-related systemic vascular atherosclerosis, often accompanied by multiple baseline comorbidities and increased potential mortality risk [20,21,22]. Impaired LVEF in patients with severe AS reflects cardiac decompensation induced by valvular obstruction and an irreversible reduction in ventricular contractility. The negative effect of low LVEF and renal impairment on mortality during the peri-procedural period has been reported [23]. These results may be explained by the fact that patients with CKD tend to be older, present with higher STS scores, and have a higher frequency of impaired LVEF [19]. A larger STJ diameter may weaken the radial force from the plane between the STJ and the ascending aorta, predisposing prosthetic valve dislodgement in the self-expanding TAVR [15,24]. Technical failure may increase all-cause mortality at 30 days and 1 year; nevertheless, the prognostic impact may be attenuated owing to the longer duration of follow-up which seems to separate immediately after the procedure but remain parallel to the 1-year trends. Similar results were found for BAV and TAV subgroups.

New generation devices feature precise implantation and external sealing to minimize peri-procedural complications. New-generation devices may improve both cardiac and vascular technical success rates owing to the presence of taller sealing skirts, smaller profile delivery systems, and the ability to reposition, in light of adverse impacts of technical failure on clinical prognosis [25,26]. Better outcomes with new-generation devices have been demonstrated in a recent meta-analysis [27]. According to the VARC-2 definition, 5.7% of patients showed major vascular complications associated with an increase in 1-year mortality [25]. Closure device failure is linked to higher vascular complications after TAVR [26]. Vascular technical failures are associated with a 1.9-fold increase in the 1-year risk of cardiovascular mortality after TAVR [8]. Therefore, the development of new closure devices may further improve clinical outcomes.

## 5. Limitations

This single-center retrospective study has several limitations that warrant consideration. First, in real-world settings, clinical patient selection was at the discretion of the operator, so selection bias could not be avoided. Some baseline characteristics, such as age and STS score, differed between bicuspid and tricuspid AS cohorts. Although technical failure was an independent predictor of 30-day and 1-year all-cause mortality in multivariate Cox regression analysis after adjusting for confounding variables (age, STS score, and BAV), randomized trials with comparable baseline characteristics between two groups are required to further verify our findings. Second, the results cannot be directly generalized to all TAVR recipients as transfemoral TAVR with early-generation self-expanding valves only were included in our analysis. Third, considering the low incidence of procedural complications, our study was underpowered to assess the association between baseline characteristics and every single component of the technical failure despite the large sample size. We did not examine the CT-scan data on peripheral vessels, which are theoretically related to vascular technical failure. Finally, patients for whom 1-year follow-up was completed were enrolled in our analysis. We cannot draw any conclusions regarding the impact of technical failure on longer-term prognostic implications between the two groups.

## 6. Conclusions

In our study, all patients were implanted with first-generation self-expanding, of which bicuspid AS accounted for 52.5% of TAVR candidates. Technical success was achieved in 82.3% of these patients according to the new VARC-3 criteria. Given the comparable technical success rate and other clinical outcomes versus tricuspid AS, TAVR is a feasible option for bicuspid AS patients. Our findings provide crucial evidence that bicuspid AS patients might benefit from TAVR.

## Figures and Tables

**Table 1:** Baseline Characteristics of Bicuspid AS and Tricuspid AS Patients.

	All *n* = 402	Bicuspid AS *n* = 211	Tricuspid AS *n* = 191	*p* Value
**Age, yrs**	**75.0 (70.0–80.0)**	**74.0 (69.0–79.0)**	**77.0 (71.0–82.0)**	**<0.001**
Male	232 (57.7)	123 (58.3)	109 (57.1)	0.804
BMI	23.1 (20.8–25.3)	22.9 (20.5–24.8)	23.1 (20.9–26.0)	0.124
**STS, %**	**4.7 (2.9–8.6)**	**4.4 (2.6–7.6)**	**5.4 (3.4–9.3)**	**0.003**
NYHA III/IV	330 (82.1)	172 (81.5)	158 (82.7)	0.753
Smoker	91 (22.6)	43 (20.4)	48 (25.1)	0.256
**Hypertension**	**232 (57.7)**	**108 (51.2)**	**124 (64.9)**	**0.005**
Diabetes	91 (22.6)	43 (20.4)	48 (25.1)	0.256
Atrial fibrillation	74 (18.4)	40 (19.0)	34 (17.8)	0.765
CKD	218 (54.2)	105 (49.8)	113 (59.2)	0.059
**PVD**	**57 (14.2)**	**19 (9.0)**	**38 (19.9)**	**0.002**
Prior PCI	43 (10.7)	18(8.5)	25 (13.1)	0.140
Prior MI	7 (1.7)	3 (1.4)	4 (2.1)	0.713
Prior pacemaker implantation	12 (3.0)	5 (2.4)	7 (3.7)	0.446
EF, %	58.8 (46.6–64.6)	58.7 (46.6–64.6)	58.9 (46.6–64.7)	0.547
EF ≤ 50%	282 (70.9)	147 (70.3)	135 (71.4)	0.811
Max velocity, m/s	4.7 (4.3–5.3)	4.8 (4.3–5.5)	4.7 (4.3–5.2)	0.056
**Mean gradient, mmHg**	53.0 (42.0–67.0)	53.0 (43.0–71.0)	50.5 (41.0–64.0)	**0.013**
**AVA, cm^2^**	0.6 (0.5–0.7)	0.6 (0.4–0.7)	0.7 (0.5–0.8)	**<0.001**
≥ moderate MR	94 (23.6)	42 (19.1)	54 (28.4)	**0.029**
≥ moderate TR	62 (15.5)	30 (14.4)	32(16.8)	0.493
Perimeter derived diameter, mm	24.5 (22.9–26.5)	24.5 (23.3–26.6)	24.4 (22.7–26.2)	0.109
**STJ diameter, mm**	**30.5 (27.6–33.3)**	**31.3 (29.1–34.1)**	**29.3 (26.2–32.2)**	**<0.001**
**STJ height, mm**	**21.8 (19.5–24.7)**	**22.1 (19.7–25.2)**	**21.6 (19.1–23.6)**	**0.017**
**AA diameter at 4 cm, mm**	**37.4 (34.6–40.6)**	**39.0 (36.3–42.0)**	**35.7 (33.1–38.8)**	**<0.001**
**Aortic root angle, degree**	**51.0 (45.0–59.0)**	**53.5 (46.8–60.0)**	**50.0 (45.0–56.0)**	**0.004**
**LM height, mm**	**14.2 (12.0–16.7)**	**14.9 (12.9–18.1)**	**13.3 (11.3–15.6)**	**<0.001**
RCA height, mm	16.4 (14.4–18.5)	16.7 (14.7–18.7)	16.2 (14.3–18.1)	0.174
**≥severe calcification**	**209 (52.3)**	**120 (57.4)**	**89 (46.6)**	**0.030**

Values are presented as *n* (%), or median (25th, 75th percentiles). *p* values in bold are statistically significant. AS = aortic stenosis; BMI = body mass index; STS = the Society of Thoracic Surgeons; NYHA = New York Heart Association; CKD = chronic kidney disease, PVD = peripheral vascular disease; PCI = percutaneous coronary intervention; MI = myocardial infarction; EF = ejection fraction; AVA = aortic valve area; MR = mitral regurgitation; TR = tricuspid regurgitation; STJ = sinotubular junction; AA = Ascending aorta; LM = left main artery; RCA = right coronary artery.

**Table 2:** Procedural characteristics and in-hospital outcomes.

	All *n* = 402	Bicuspid AS *n* = 211	Tricuspid AS *n* = 191	*p* Value
Pre-dilatation	396 (98.5)	210 (99.5)	186 (97.4)	0.077
**Post-dilatation**	**249 (61.9)**	**144 (68.2)**	**113 (55.0)**	**0.006**
Conversion to surgery	5 (1.2)	4 (1.9)	1 (0.5)	0.375
Coronary obstruction	7 (1.7)	2 (0.9)	5 (2.6)	0.264
Aortic dissection	7 (1.7)	5 (2.4)	2 (1.0)	0.453
Annular rupture	2 (0.5)	1 (0.5)	1 (0.5)	1.000
In-hospital mortality	9 (2.2)	6 (2.8)	3 (1.6)	0.450
Myocardial infarction	3 (0.7)	2 (0.9)	1 (0.5)	0.622
Stroke	6 (1.5)	5 (2.4)	1 (0.5)	0.219
Disabling stroke	2 (0.5)	1 (0.5)	1 (0.5)	1.000
Non-disabling stroke	4 (1.0)	4 (1.9)	0 (0)	0.125
New atrial fibrillation	18 (4.5)	7 (3.3)	11 (5.8)	0.237
**Pacemaker implantation**	**42 (10.4)**	**14 (6.6)**	**28 (14.7)**	**0.009**
Second valve implantation	46 (11.4)	23 (10.9)	23 (12.0)	0.720
Severe PPM	23(5.9)	13 (6.4)	10 (5.3)	0.649
Technical success	331(82.3)	174 (82.5)	157 (82.2)	0.944
Cardiac success	349 (86.8)	183 (86.7)	166 (86.9)	0.957
Vascular success	379 (94.3)	180 (94.2)	180 (94.3)	0.975

Values are presented as *n* (%). *p* values in bold are statistically significant. PPM = patient-prosthesis mismatch.

**Table 3:** Clinical outcomes during 30-day and 1-year follow-up.

	All *n* = 402	Bicuspid AS *n* = 211	Tricuspid AS *n* = 191	*p* Value
30-day clinical outcomes				
Mortality	9 (2.2)	5 (2.4)	4 (2.1)	1.000
Cardiovascular	7 (1.7)	4 (1.9)	3 (1.6)	1.000
Non-cardiovascular	1 (0.5)	1 (0.5)	1 (0.5)	1.000
NYHA III/IV	109 (28.7)	45 (22.5)	63 (35.6)	0.005
Myocardial infarction	4 (1.0)	3 (1.4)	1(0.5)	0.625
Life-threatening bleeding	14 (3.5)	9 (4.3)	5 (2.6)	0.368
Pacemaker implantation	48 (12.0)	14 (6.7)	34 (17.8)	0.001
1-year clinical outcomes				
Mortality	31 (7.7)	11 (5.2)	20 (10.5)	0.048
Cardiovascular	21 (5.2)	7 (3.3)	14 (7.3)	0.071
Non-cardiovascular	10 (2.5)	4 (1.9)	6 (3.1)	0.631

Values are *n* (%). NYHA = New York Heart Association.

**Table 4:** Reasons for Technical Failure.

	All *n* = 402	Bicuspid AS *n* = 211	Tricuspid AS *n* = 191	*p* Value
Procedural death	2 (0.5)	2 (0.9)	0	0.500
Cardiac reason				
Conversion to surgery	5 (1.2)	4 (1.9)	1(0.5)	0.375
Pericardial drainage due to annular rupture	1 (0.2)	1 (0.5)	0	1.000
PCI due to coronary obstruction	4 (1.0)	1 (0.5)	3 (1.6)	0.350
Second valve implantation	46 (11.4)	23 (10.9)	23 (12.0)	0.720
Valve dislocation/embolization	9 (2.2)	6 (2.8)	3 (1.6)	0.508
Valve retrieval	0	0	0	-
Vascular reason (vascular/access-related complication)				
Stent placement	5 (1.2)	2 (0.9)	3 (1.6)	0.672
Vascular surgery	18 (4.5)	10 (4.8)	8 (4.2)	0.782

Values are presented as *n* (%). SAVR = surgical aortic valve replacement; PPM = patient-prosthesis mismatch; PCI = percutaneous coronary intervention.

**Table 5:** The Adjusted Association of the Relevant Variables with technical success and cardiac technical success, Using Logistic Regression analysis using a Likelihood Ratio Method.

Variables	Technical Failure				Cardiac Technical Failure			
	Univariate		Multivariate		Univariate		Multivariate	
	β (SE)	*p* Value	β (SE)	*p* Value	β (SE)	*p* Value	β (SE)	*p* Value
Age, yrs	0.025	0.201	-	-	0.020	0.351	-	-
BAV	−0.018	0.944	-	-	0.016	0.957	-	-
Male	0.143	0.592	-	-	**0.705**	**0.029**	-	-
BMI	−0.056	0.149	-	-	0.062	0.159	-	-
STS	0.026	0.191	-	-	0.031	0.137	-	-
NYHA III/IV	0.085	0.807	-	-	0.408	0.341	-	-
CKD	**0.758**	**0.007**	**0.767**	**0.007**	**0.764**	**0.016**	**0.702**	**0.035**
Hypertension	−0.345	0.189	-	-	**−0.490**	**0.098**	-	-
Diabetes	−0.107	0.737	-	-	−0.127	0.725	-	-
PVD	−0.314	0.440	-	-	−0.519	0.293	-	-
EF ≤ 50%	**0.626**	**0.023**	**-**	**-**	**1.088**	**<0.001**	**0.881**	**0.005**
≥ moderate MR	**0.492**	**0.090**	**-**	**-**	**0.736**	**0.020**	-	-
≥ moderate TR	0.381	0.259	-	-	**0.707**	**0.047**	-	-
Perimeter derived diameter, mm	0.053	0.287	-	-	**0.173**	**0.002**	-	-
STJ diameter ≥ 31.0 mm	**0.539**	**0.040**	**0.661**	**0.015**	**1.019**	**0.001**	**1.091**	**0.001**
STJ height, mm	0.028	0.373	-	-	**0.069**	**0.045**	-	-
AA diameter at 4 cm, mm	0.002	0.941	-	-	0.043	0.164	-	-
AA diameter ≥ 40 mm (at 4 cm)	0.184	0.518			0.431	0.166		
≥ severe calcification	0.067	0.802	-	-	0.213	0.481	-	-

Variables with a *p*-value <0.10 in univariate logistics regression analysis were included in the multivariate model. Univariate variables in bold are statistically and clinically significant. BAV = bicuspid aortic valve stenosis; BMI = body mass index; STS = the Society of Thoracic Surgeons; NYHA = New York Heart Association; CKD = chronic kidney disease; PVD = peripheral vascular disease; EF = ejection fraction; MR = mitral regurgitation; TR = tricuspid regurgitation; STJ = sinotubular junction; AA = ascent aorta. Factors included in multivariate regression are shown in bold.

**Table 6:** Multivariate Cox regression analysis of 30-day all-cause mortality, using a Enter Method.

	Multivariable Regression	
	β (SE)	*p* Value
BAV	0.385	0.616
Age	0.035	0.600
**STS**	**0.117**	**<0.001**
**Technical failure**	**1.487**	**0.032**
CKD	1.303	0.244
**STJ diameter ≥ 31.0 mm**	**2.466**	**0.018**

Variables with a *p*-value < 0.10 in univariate logistics regression analysis were included in the multivariate model. Univariate variables in bold are statistically and clinically significant. BAV = bicuspid aortic valve stenosis; STS = the Society of Thoracic Surgeons; CKD = chronic kidney disease; STJ = sinotubular junction; Factors included in multivariate regression are shown in bold.

**Table 7:** Multivariate Cox regression analysis of one-year all-cause mortality, using a Enter Method.

	Multivariable Regression	
	β (SE)	*p* Value
BAV	−0.392	0.317
Age	−0.018	0.567
**STS**	**0.110**	**<0.001**
**Technical failure**	**0.915**	**0.021**
EF ≤ 50%	0.413	0.337
CKD	0.984	0.061
≥ moderate MR	0.633	0.107

Variables with a *p*-value < 0.10 in univariate logistics regression analysis were included in the multivariate model. Univariate variables in bold are statistically and clinically significant. BAV = bicuspid aortic valve stenosis; STS = the Society of Thoracic Surgeons; CKD = chronic kidney disease; STJ = sinotubular junction; Factors included in multivariate regression are shown in bold.

## Data Availability

The data presented in this study are available on request from the corresponding author. The data are not publicly available due to privacy restrictions.

## Abbreviations

VARC-3	Valve Academic Research Consortium
AS	aortic stenosis
CKD	chronic kidney disease
STJ	sinotubular junctional diameter
LVEF	left ventricular ejection fraction
TAVR	transcatheter aortic valve replacement
MDCT	multi-detected computed tomography
IQR	interquartile range
